# Clinical, Laboratory, and Imaging Characteristics of Liver Involvement in Hospitalized Children With COVID‐19 in Iran During 2020–2021: A Cross‐Sectional Study

**DOI:** 10.1002/hsr2.72859

**Published:** 2026-07-25

**Authors:** Shahnaz Armin, Roxana Mansour Ghanaiee, Abdollah Karimi, Mahzad Rahmani, Ahmad Reza Shamshiri, Leila Azimi, Maryam Rajabnejad, Hannan Khodaei, Ahmadmoeen Karimi, Seyede Mahsan Hoseini‐Alfatemi, Yasna Soleimani

**Affiliations:** ^1^ Pediatric Infections Research Center Research Institute for Children's Health, Shahid Beheshti University of Medical Sciences Tehran Tehran Province Iran; ^2^ Dental Research Center Dentistry Research Institute, Tehran University of Medical Sciences Tehran Tehran Province Iran; ^3^ Department of Environmental Health Engineering School of Public Health, Tehran University of Medical Sciences Tehran Tehran Province Iran

**Keywords:** children, COVID‐19, hospitalized, liver, pediatrics

## Abstract

**Background and Aims:**

Coronavirus disease 2019 (COVID‐19) affects multiple organs, including the liver. Although pediatric patients generally experience milder disease, liver involvement has been increasingly reported, and its impact on outcomes remains unclear. This study aimed to evaluate clinical, laboratory, and imaging characteristics of liver involvement in hospitalized children with COVID‐19.

**Methods:**

We retrospectively analyzed 301 pediatric patients with confirmed COVID‐19 admitted to Mofid Children's Hospital, Tehran, Iran, from March 2020 to July 2021 who met the inclusion criteria. Patients were divided into two groups based on liver involvement. Liver involvement was defined as an alanine aminotransferase (ALT) level > 40 IU/L. Variables were compared between groups with and without liver involvement using both univariate and multivariable logistic regression analyses. A *p* < 0.05 was considered statistically significant.

**Results:**

Elevated ALT was observed in 84 patients (27.9%). Liver involvement occurred significantly more often in children aged 10 years and older. Patients with hepatic involvement had higher rates of leukopenia, thrombocytopenia, elevated blood urea nitrogen (BUN), tachycardia, hypoxia, pulmonary infiltrations, and shock. The mean hospital stay was longer (7.27 vs. 6.07 days, *p* = 0.107). Intensive care unit (ICU) admission (29.7% vs. 15%; *p* = 0.003) and mortality (14.3% vs. 3.7%, *p* < 0.001) were both higher in the liver involvement group. Multivariate analysis showed that in children under 10 years of age, elevated BUN and pulmonary infiltrations independently predicted liver involvement, while in children ≥ 10 years, pre‐admission antibiotic use and normal hemoglobin (Hb) were associated factors with hepatic involvement.

**Conclusion:**

Liver involvement is common among hospitalized pediatric COVID‐19 patients and is associated with worse clinical outcomes, including higher ICU admission and mortality rates. Early recognition and monitoring of hepatic dysfunction may improve management strategies in this population.

## 1 Background

Coronavirus disease 2019 (COVID‐19), caused by severe acute respiratory syndrome Coronavirus‐2 (SARS‐CoV‐2), became a major global health concern after its discovery in Wuhan, China, in late 2019 [1, 2]. Initially, it was thought to be a respiratory disease, but now it is known that the virus can affect multiple organs, including the gastrointestinal tract, kidneys, cardiovascular system, and particularly the liver [3, 4, 5].

Children generally have a lower risk of developing severe cases of COVID‐19 compared to adults and usually show less severe symptoms. However, some children have been reported to experience multi‐organ involvement that required hospitalization [6, 7]. Among these, liver involvement has gained increasing attention due to its potential impact on disease severity and patient outcomes [8]. Reports indicate that 10%–30% of pediatric COVID‐19 patients may show abnormal liver enzymes, ranging from mild, transient elevation to more significant dysfunction, especially in those with multisystem inflammatory syndrome in children (MIS‐C) [9, 10].

Liver injury in COVID‐19 can occur through both direct viral effects and indirect systemic mechanisms. Direct effects happen when the virus binds to angiotensin‐converting enzyme 2 (ACE2) receptors on liver cells, allowing the virus to enter these cells. Indirect mechanisms include immune‐mediated injury, hypoxia, MIS‐C‐associated hepatic dysfunction, and drug‐induced hepatotoxicity [11].

Common indicators of liver involvement in COVID‐19 include elevated aspartate aminotransferase (AST), alanine aminotransferase (ALT), gamma‐glutamyl transferase (GGT), and alkaline phosphatase (ALP), along with decreased albumin levels. Although AST is most frequently elevated, it is not unique to the liver, as it can also be found in the heart and muscle tissues. In contrast, ALT is more specific to the liver and serves as a better marker of liver damage, particularly in children [11, 12].

This study aimed to comprehensively evaluate the clinical, laboratory, and imaging features of liver involvement in hospitalized pediatric patients with COVID‐19, and to identify factors associated with hepatic injury and adverse outcomes in this population.

## 2 Methods

### 2.1 Study Design

Children aged 18 years or younger who were admitted to Mofid Children's Hospital, a referral center for treating pediatric patients with COVID‐19 in Tehran, Iran, between March 2020 and July 2021 were retrospectively analyzed. Total admitted cases were 311 patients.

Inclusion criteria were a positive COVID‐19 polymerase chain reaction (PCR) test during the first 3 days of admission and available liver function test results. Exclusion criteria were the absence of the above‐mentioned conditions as well as a history of chronic liver disease. Ten individuals with a past medical history of hepatic disease were excluded from the research, and ultimately, 301 patients were enrolled in our study.

Data were collected by reviewing medical records using a structured questionnaire developed based on the World Health Organization (WHO) COVID‐19 Case Report Form [13]. Extracted variables contained demographic features, comorbidities, clinical symptoms, vital signs, laboratory and imaging results, pre‐admission and in‐hospital medications, and outcomes such as ICU admission and discharge status.

Age‐specific definitions of tachycardia, tachypnea, and hypotension were adopted from standard pediatric references values reported in the Nelson Textbook of Pediatrics [14] (Table S1).

### 2.2 Patient Classification

Patients were classified as two groups based on the presence or absence of liver involvement. Liver involvement was defined as ALT > 40 IU/L, a commonly used threshold in pediatric studies assessing hepatic injury in COVID‐19 and other viral infections. This cutoff is consistent with standard pediatric upper limits reported in prior literature and in the Nelson Textbook of Pediatrics [14].

ALT was chosen as the primary marker of hepatic involvement because it is more specific to the liver, whereas AST can be elevated in a variety of non‐hepatic conditions, including muscle injury [11, 12]. GGT and bilirubin were not used for classification as they are less sensitive for detecting mild hepatic involvement in acute pediatric COVID‐19 and were also not consistently available in this retrospective dataset.

### 2.3 Statistical Analysis

IBM SPSS Statistics version 27.0. was used for data analysis. Categorical variables were presented as counts and percentages, while continuous variables as mean ± standard deviation (SD). Comparisons between groups were performed using Chi‐square test, Fisher's exact test, independent samples *T* test, and logistic regression. Analyses were performed to identify factors associated with liver involvement. Variables with *p *< 0.20 in univariate analysis, along with clinically important variables, were entered into the multivariate logistic regression model to avoid exclusion of potential important confounders [15]. Because age showed a significant interaction with liver involvement, separate multivariable models were performed for patients aged < 10 and ≥ 10 years. A two‐sided *p* < 0.05 was considered statistically significant.

## 3 Results

### 3.1 Demographic and Epidemiological Characteristics

A total of 301 pediatric patients who met the inclusion criteria were enrolled in our study. Among them, 84 (27.9%) had elevated ALT levels (> 40 IU/L), defined as liver involvement, while 217 (72.1%) had normal liver enzymes. In the liver involvement group, 51 (60.7%) patients were male compared with 123 (56.7%) in the normal ALT group (*p* = 0.53). The average age of the participants was 6.23 ± 5.23 years. In statistical analysis based on age groups, there was a significant association between liver injury and patients aged 10 years and older (*p* < 0.001) (Table 1).

**Table 1:** Comparison of age groups between children with and without liver involvement in COVID‐19.

Age group	No liver involvement (*n* = 217)	Liver involvement (*n* = 84)	Total (*n* = 301)	*p*‐value
0–12 months	6 (85.7%)	1 (14.3%)	7 (100.0%)	0.005
1–5 years	117 (78.5%)	32 (21.5%)	149 (100.0%)	0.006
5–10 years	42 (75.0%)	14 (25.0%)	56 (100.0%)	0.044
≥ 10 years	52 (58.4%)	37 (41.6%)	89 (100.0%)	Ref.

*Note:* Among all underlying comorbidities, immunodeficiency and chronic neurological diseases were more common in the group with liver involvement—−16.7% versus 15.2%; *p* = 0.754 for immunodeficiency, and 8.3% versus 7.4%; *p* = 0.779 for neurologic disease. Other underlying comorbidities included autoimmune diseases, chronic cardiac disease, asthma, and chronic pulmonary disease, with no significant differences between the two groups.

### 3.2 Signs and Symptoms

Of all the symptoms included in our questionnaire, fever was the most common complaint among participants with impaired liver transaminases, with an incidence of 71.4%. This was followed, in order of prevalence, by cough, shortness of breath, muscle ache, and fatigue. However, symptoms did not differ significantly between groups.

An assessment of vital signs upon admission between two groups of liver‐involved and non‐involved patients showed that only temperature > 38°C was more frequent in the non‐involved group (18.9% vs. 14.3%). In contrast, all other vital sign abnormalities were more common in the liver‐involved group: (40.2% vs. 27.4% for tachycardia, 17.7% vs. 15.9% for tachypnea, 7.1% vs. 3.2% for hypotension, and 15.8% vs. 6.9% for hypoxia). Among these, tachycardia (*p* = 0.026) and hypoxia (*p* = 0.025) were statistically significant.

### 3.3 Laboratory and Imaging Findings

In complete blood count (CBC) analysis, reduced hematocrit (HCT), leukopenia, neutropenia, and thrombocytopenia were more common in patients with elevated liver enzymes, whereas anemia and leukocytosis were more frequent among patients with normal transaminase levels.

The most common coagulation abnormalities overall were increased international normalized ratio (INR) (25.2%), prolonged partial thromboplastin time (PTT) (18.6%), and prolonged prothrombin time (PT) (14.8%). Prolonged PTT was observed more frequently in liver‐impaired patients, while alterations in INR and PT were relatively more common in the group with normal liver enzymes.

A notable 84.1% of patients with abnormal level of ALT also had high AST, compared to 35.3% in the normal ALT group. Elevated total bilirubin, BUN, creatinine, erythrocyte sedimentation rate (ESR), and creatine phosphokinase (CPK) were more frequent in those with abnormal transaminase. Moreover, pulmonary infiltrations were more prevalent in patients with liver impairment (60%) than in those without (38%).

Among all laboratory tests and imaging, statistically significant associations were found for leukopenia (*p* = 0.049), thrombocytopenia (*p* = 0.012), elevated BUN (*p* = 0.017), and pulmonary infiltrations (*p *= 0.003).

### 3.4 Pre‐Admission and In‐Hospital Treatments

The most used medications before admission were antibiotics, followed by corticosteroids, with no significant differences between groups. The most frequently prescribed treatments on the first day of admission included antibiotics (69%), corticosteroids (39%), antivirals (29.5%), oxygen therapy (20%), and blood‐derived products (12%). Significant differences between patients with and without liver enzyme impairment were only observed in the use of anticoagulants on the first day of admission (*p* < 0.001).

### 3.5 Complications and Outcomes

The most common complications during hospitalization were lower respiratory tract infection (11%), upper respiratory tract infection (8%), shock (5.6%), acute kidney injury (AKI) (5.6%), and liver dysfunction (4.6%). Among all complications, shock occurred significantly more often in patients with abnormal ALT (*p* = 0.023).

The average hospital stay was longer in the liver involvement group (7.27 ± 5.23 vs. 6.07 ± 5.92 days), although the difference was not statistically significant (mean difference = 1.22 days, 95% CI: −0.26 to 2.71; *p *= 0.107). ICU admission rate was significantly higher (29.7% vs. 14.7%, OR = 2.450, 95% CI: 1.345–4.462; *p *= 0.003), and mortality was also significantly greater (14.3% vs. 3.7%, OR = 4.354, 95% CI: 1.711‐11.078; *p *< 0.001). Notably, 48% of patients with liver involvement who required ICU admission eventually experienced a fatal outcome.

## 4 Multivariate Logistic Regression

Variables with *p* < 0.20 in univariate analysis, along with clinically important variables, were entered into the multivariate logistic regression model (Table 2).

**Table 2:** Variables included in the multivariate logistic regression model.

Variable	No liver involvement (*n* = 217)	Liver involvement (*n* = 84)	*p*‐value
Age ≥ 10 years	52 (58.4%)	37 (41.6%)	< 0.001
Tachycardia	59 (27.4%)	34 (40.2%)	0.026
Hypoxia	15 (6.9%)	13 (15.4%)	0.025
Pre‐admission antibiotics	39 (17.9%)	23 (27.3%)	0.07
1st day of admission anticoagulants	13 (6.0%)	18 (21.4%)	< 0.001
1st day of admission antivirals	58 (26.7%)	31 (37.0%)	0.08
O_2_ therapy	38 (17.5%)	23 (27.3%)	0.06
Leukopenia	50 (23.0%)	30 (35.7%)	0.049
Thrombocytopenia	39 (18.0%)	27 (32.1%)	0.012
Hemoglobin ≥ 11	111 (51.1%)	48 (57.1%)	0.35
BUN > 20	14 (6.4%)	13 (15.4%)	0.017
Infiltration	64 (29.5%)	40 (47.6%)	0.003
Shock	8 (3.7%)	9 (10.7%)	0.023
ICU admission	32 (14.7%)	25 (29.7%)	0.003
Mortality	8 (3.7%)	12 (14.3%)	< 0.001

In multivariate logistic regression analysis, among children under 10 years, elevated BUN and pulmonary infiltrations independently predicted liver involvement. In children aged 10 years and older, pre‐admission antibiotic use and normal hemoglobin level were independently associated with liver involvement (Table 3).

**Table 3:** Significant independent predictors of liver involvement in multivariate logistic regression analysis.

Age group	Variable	OR	95% CI	*p*‐value
< 10 years	BUN ≥ 20	3.48	1.26–9.58	0.02
	Pulmonary infiltrations	2.02	1.04–4.04	0.046
≥ 10 years	Pre‐admission antibiotic use	26.87	3.20–225.32	0.002
	Hemoglobin ≥ 11	17.83	2.47–128.65	0.004

Abbreviations: CI, confidence interval; OR, odds ratio.

## 5 Discussion

In this study, about one‐third (27.9%) of hospitalized children with COVID‐19 had elevated ALT levels, indicating liver involvement. This prevalence is consistent with previous pediatric studies, reporting abnormal liver enzymes in 25%–33% of children [16, 17]. Liver involvement was more common in children aged older than 10 years, aligning with findings by Perez et al., who described greater hepatic injury in older children [4]. These results suggest that age‐dependent mechanisms, such as differences in immune responses and metabolic factors may contribute to hepatic injury.

Our analysis found that systemic abnormalities such as tachycardia, hypoxia, leukopenia, thrombocytopenia, and elevated BUN were significantly associated with liver injury, in agreement with Pavelescu et al. and Singh et al. [18, 19]. Previous research has also shown that liver injury is more common in children with severe systemic presentations and MIS‐C [20, 21, 22]. Together with our results, this supports that hepatic injury in pediatric COVID‐19 often reflects systemic inflammation and multi‐organ dysfunction rather than isolated hepatic damage [23, 24].

Notably, the higher frequency of reduced hematocrit in the liver involvement group, despite a greater prevalence of anemia among patients with normal transaminase levels, suggests that reduced hematocrit and anemia are not synonymous findings. Hematocrit may be affected by factors other than anemia, including alterations in plasma volume and systemic inflammation associated with liver involvement during COVID‐19.

We also found that normal hemoglobin levels were independently associated with liver involvement in children aged 10 years and older, although this finding should be interpreted cautiously given the wide confidence interval. This contrasts with studies such as Faghih Dinevari et al., which linked anemia with worse COVID‐19 outcomes [25]. A possible explanation is that children with higher hemoglobin levels might have a stronger inflammatory response, leading to greater hepatic involvement. Additionally, older children generally have a more mature immune system and higher metabolic activity, contributing to an exaggerated hepatic reaction.

Radiologically, our data showed that pulmonary infiltrations were significantly more frequent among those with liver involvement, similar to Tazarghi et al. [26]. However, Sayyahfar et al. reported that elevated liver enzymes did not reliably predict lung involvement [27]. Taken together, these findings suggest that although hepatic and pulmonary involvement often coexist, elevated liver enzymes alone may not accurately reflect the severity of respiratory compromise [28].

Medication exposure also appeared to play an important role in our study. Pre‐admission antibiotic use and in‐hospital anticoagulant treatment were significantly more common in the liver involvement group. Most studies, including those by Jinda et al., Kazemian et al., have highlighted the hepatotoxic potential of medications, particularly antiviral agents used in treatment of COVID‐19 [29, 30]. However, in our study, liver enzymes were evaluated before the initiation of in‐hospital therapy, suggesting that patients with elevated ALT levels were in a more severe clinical condition and therefore required more intensive treatment.

In terms of outcomes, liver involvement was a strong predictor of disease severity. Children with abnormal ALT levels had higher rates of ICU admission, longer hospital stays, a greater risk of shock, and significantly higher mortality rates. These findings are consistent with those of Mortazavi et al. and Abrams et al. [31, 32]. Overall, liver involvement in pediatric COVID‐19 appears to be multifactorial, age‐dependent, and closely related to systemic illness and adverse outcomes. This research gives an alarming sign to physicians to be more cautious when managing children with COVID‐19 and abnormal ALT levels, in order to improve patient prognosis and anticipate complications.

This study has several strengths, including a relatively large cohort of pediatric patients with positive COVID‐19 PCR and thorough evaluation of clinical, laboratory, and imaging factors. Limitations include the single‐center nature of the study, its retrospective design, the unavailability of a complete liver function panel for all patients, particularly GGT and bilirubin measurements, as well as the lack of baseline liver enzyme data prior to hospital admission. Data on potentially hepatotoxic medications used before hospitalization, especially in patients with comorbidities, were not consistently available therefore and could not be analyzed. Additionally, some underlying comorbidities may have acted as potential confounders but were not included in the multivariate model due to their low prevalence. Furthermore, symptoms specifically suggestive of hepatic dysfunction, such as jaundice, right upper quadrant pain, dark urine, and pale stools, were not systematically recorded in the medical files and could not be evaluated.

Future studies should be multicenter and prospective in design, with comprehensive serial liver function assessments and baseline hepatic status evaluations to better distinguish pre‐existing liver abnormalities from COVID‐19‐related hepatic involvement and to clarify the long‐term hepatic outcomes and underlying mechanism of liver injury in pediatric population.

## 6 Conclusion

This study demonstrates that liver involvement is a common and clinically significant finding among hospitalized pediatric COVID‐19 patients. It is closely linked to older age, systemic inflammation, and adverse outcomes, with higher risk of ICU admission, prolonged hospitalization, and mortality. Routine monitoring of liver function tests, especially in children with multi‐organ involvement, may aid proper risk assessment and clinical decision‐making. Future pediatric‐specific studies are necessary to clarify underlying mechanisms and to establish preventive and therapeutic strategies for COVID‐19‐related liver injury.

## Author Contributions

**Shahnaz Armin:** supervision, writing – review and editing, project administration. **Roxana Mansour Ghanaiee:** supervision; writing – review and editing. **Abdollah Karimi:** supervision, writing – review and editing, conceptualization. **Mahzad Rahmani:** data curation, software. **Ahmad Reza Shamshiri:** software, formal analysis, investigation. **Leila Azimi:** data curation. **Maryam Rajabnejad:** data curation, software. **Hannan Khodaei:** data curation, software. **Ahmadmoeen Karimi:** visualization, data curation. **Seyede Mahsan Hoseini‐Alfatemi:** methodology, resources, writing – original draft, writing – review and editing, validation, conceptualization, supervision. **Yasna Soleimani:** writing – review and editing, writing – original draft, resources, software, investigation, visualization; validation; supervision. All authors have read and approved the final version of the manuscript. Seyede Mahsan Hoseini‐Alfatemi had full access to all study data and took responsibility for the integrity of the data and the accuracy of the data analysis.

## Funding

The authors have nothing to report.

## Ethics Statement

This study was reviewed and approved by the Ethics Committee of the Research Institute for Children's Health at Shahid Beheshti University of Medical Sciences (ethical code: IR.SBMU.RICH.REC.1402.014).

## Conflicts of Interest

The authors declare no conflicts of interest.

## Transparency Statement

Shahnaz Armin affirms that this manuscript is an honest, accurate, and transparent account of the study being reported; that no important aspects of the study have been omitted, and that any discrepancies from the study as planned have been explained.

## Supporting information


**Table S1:** Age‐specific definitions of vital sign abnormalities.

## Data Availability

The data supporting the findings of this study are available from the corresponding authors upon request.
