# Supplementary material for: Clinical, Laboratory, and Imaging Characteristics of Liver Involvement in Hospitalized Children With COVID‐19 in Iran During 2020–2021: A Cross‐Sectional Study
Source: Health Sci Rep. 2026 Jul 25;9(8):e72859. doi: 10.1002/hsr2.72859 (PMC13401695; doi:10.1002/hsr2.72859)
Supplement: Supplementary file 1 — Table S1: Age‐specific definitions of vital sign abnormalities. [file HSR2-9-e72859-s001.docx]

Supplementary Table S1. Age-specific definitions of vital sign abnormalities

| Age group | Tachycardia  (beats/min) | Tachypnea  (breaths/min) | Hypotension  (SBP, mmHg) |
| --- | --- | --- | --- |
| <28 days | >160 | >60 | <60 |
| 1-12 months | >140 | >40 | <70 |
| 1-3 years | >130 | >40 | <70 + (2 × age) |
| 3-5 years | >120 | >40 | <70 + (2 × age) |
| 5-10 years | >100 | >30 | <70 + (2 × age) |
| >10 years | >100 | >30 | <90 |

- Fever: Forehead temperature >38°C measured using a digital infrared thermometer.
- Hypoxemia: Oxygen saturation (SpO2)▒<▒92%.
